# The Role of Neurotrophic Factors Conjugated to Iron Oxide Nanoparticles in Peripheral Nerve Regeneration: *In Vitro* Studies

**DOI:** 10.1155/2014/267808

**Published:** 2014-07-16

**Authors:** Ofra Ziv-Polat, Abraham Shahar, Itay Levy, Hadas Skaat, Sara Neuman, Federica Fregnan, Stefano Geuna, Claudia Grothe, Kirsten Haastert-Talini, Shlomo Margel

**Affiliations:** ^1^NVR Research Ltd., Heharash Street 11, 74031 Ness-Ziona, Israel; ^2^Institute of Nanotechnology and Advanced Materials, Department of Chemistry, Bar-Ilan University, 52900 Ramat-Gan, Israel; ^3^Department of Clinical and Biological Sciences, Università degli studi di Torino, Regione Gonzole 10, 10043 Orbassano, Italy; ^4^Institute of Neuroanatomy, Hannover Medical School, Carl-Neuberg-Street 1, 30623 Hannover, Germany; ^5^Center for Systems Neuroscience (ZSN) Hannover, 30559 Hannover, Germany

## Abstract

Local delivery of neurotrophic factors is a pillar of neural repair strategies in the peripheral nervous system. The main disadvantage of the free growth factors is their short half-life of few minutes. In order to prolong their activity, we have conjugated to iron oxide nanoparticles three neurotrophic factors: nerve growth factor (*β*NGF), glial cell-derived neurotrophic factor (GDNF), and basic fibroblast growth factor (FGF-2). Comparative stability studies of free versus conjugated factors revealed that the conjugated neurotrophic factors were significantly more stable in tissue cultures and in medium at 37°C. The biological effects of free versus conjugated neurotrophic factors were examined on organotypic dorsal root ganglion (DRG) cultures performed in NVR-Gel, composed mainly of hyaluronic acid and laminin. Results revealed that the conjugated neurotrophic factors enhanced early nerve fiber sprouting compared to the corresponding free factors. The most meaningful result was that conjugated-GDNF, accelerated the onset and progression of myelin significantly earlier than the free GDNF and the other free and conjugated factors. This is probably due to the beneficial and long-acting effect that the stabilized conjugated-GDNF had on neurons and Schwann cells. These conclusive results make NVR-Gel enriched with conjugated-GDNF, a desirable scaffold for the reconstruction of severed peripheral nerve.

## 1. Introduction

In case of peripheral nerve injuries with long distance defects (gaps), autologous nerve grafting is the treatment of choice. However, the availability of autologous nerve grafts is limited especially when a large amount of grafting material is needed, because it requires sacrifices of healthy nerves [1]. Therefore, many efforts are being made to develop artificial nerve grafts. The artificial nerve grafts are usually composed of conduits, which guide the regenerating axons to the distal nerve stump, filled with biomaterials decorated with neurotrophic factors (neuronal growth factors) [2].

Neurotrophic factors are signaling proteins which support neural survival and axonal growth. In addition, neurotrophic factors play a significant role in the maintenance of neuronal function throughout an entire lifetime. There are a variety of neurotrophic factors that have been identified and studied at various levels from molecular interactions to macroscopic tissue responses (reviewed in [3–6]). In the present study we focused on three neurotrophic factors: nerve growth factor (*β*NGF), glial cell-derived neurotrophic factor (GDNF), and basic fibroblast growth factor (FGF-2).

NGF (discovered in the early 1950s by Rita Levi-Montalcini who won the 1986 Nobel Prize in Medicine for this discovery [7]) is a polypeptide composed of three subunits alpha, beta, and gamma. Only the beta subunit (*β*NGF) is responsible for the physiological activity ascribed to NGF, and it is active in the absence of the other two subunits [8]. *β*NGF is vital for the development and maintenance of both central and peripheral nervous system neurons. It is known to regulate the growth and differentiation of sympathetic and sensory neurons. In addition, *β*NGF plays a key role in nerve injury repair and in preventing neuronal degeneration (reviewed in [3, 9]).

GDNF has been demonstrated to enhance the survival and outgrowth of motor neurons as well as sensory neurons [3]. It also promotes* in vivo* myelination of unmyelinated nerve fibers [10, 11]. This factor was also found to act on dopaminergic neurons in the substantia nigra, whose death is responsible for Parkinson's disease [12, 13].

FGF-2 is a potent mitogenic polypeptide which induces cell divisions in a variety of mesodermal cell types and cells of neuroectodermal origin (reviewed in [14, 15]). FGF-2 has been shown to promote neuron survival, spinal cord regeneration, peripheral nerve regeneration, and neuritogenesis of motor neurons [16–19]. FGF-2 also stimulates the proliferation and differentiation of neuronal precursor cells from mouse and human olfactory epithelium [3, 14, 20–22] as well as of Schwann cells [23].

Because of their positive effect on neurons, neurotrophic factors are attractive candidates for therapy of acute injuries to the nervous system (such as reconstruction of peripheral nerve after a severe segment loss, especially in cases of a long distance nerve defect) as well as chronic neurodegenerative diseases (such as Alzheimer's, Huntington's, and Parkinson's disease) (reviewed in [3, 4]). Also, in laboratory research, it is common to add neurotrophic factors to cell cultures to enhance neuronal cell regeneration. However, the main disadvantage of free growth factors is their short* in vivo* half-life time due to rapid enzymatic degradation, which leads to the loss of their biological activity after a short period of time [24]. For example, the half-life time of FGF-2, brain derived neurotrophic factor (BDNF), and *β*NGF in blood is 1.5–3, 10, and 30 min, respectively [25–29]. In order to prolong their activity and bioavailability, it has been suggested to conjugate the growth factors onto or encapsulated within nanostructures of diverse types of synthetic and natural materials [24, 30].

In the present study, iron oxide nanoparticles were used for neurotrophic factor conjugation. These nanoparticles provide magnetic properties, high surface-area-to-volume ratio, biocompatibility, and biodegradability. Iron oxide magnetic nanoparticles are considered to be nontoxic and are already being used for various biomedical applications, such as diagnostics, cell labeling and sorting, DNA separation, MRI and X-ray contrast agents, gene and drug delivery, and hyperthermia (reviewed in [21, 22]). The magnetic properties of these nanoparticles may also be used for targeting of a drug immobilized on magnetic materials under the action of an external magnetic field [31]. In addition, the magnetic properties of the iron oxide nanoparticles enable their purification from excess reagents, during the synthesis process, easily by using magnetic columns.

The aim of the present study was to examine* in vitro* the stability and the biological effects of *β*NGF, GDNF, and FGF-2 conjugated covalently to iron oxide nanoparticles, with the prospective to use them in artificial conduits for peripheral nerve reconstruction. Our studies were conducted on cultures of organotypic dorsal root ganglion (DRG) seeded in NVR-Gel [32] as a possible hydrogel scaffold for filling artificial conduit [2].

## 2. Materials and Methods

### 2.1. Synthesis of Iron Oxide Nanoparticles

Magnetic iron oxide nanoparticles were prepared in two main ways as follows.

- Gelatin coated iron oxide nanoparticles containing the fluorescent probe rhodamine of 15.0 ± 1.4 nm dry diameter were prepared by nucleation and then controlled the growth of iron oxide layers onto rhodamine isothiocyanate (RITC) conjugated gelatin/iron oxide nuclei, according to our previous publications [21, 33].
- Dextran coated iron oxide magnetic nanoparticles of 9.5 ± 0.9 nm dry diameter were prepared as described previously by Molday and Mackenzie [34].

### 2.2. Conjugation of Neural Growth Factors to the Iron Oxide Nanoparticles

Covalent conjugation of *β*NGF, GDNF, or FGF-2 (PeproTechAsia, Israel) to the surface of the iron oxide nanoparticles (each type individually) was performed as described previously [21, 22, 33]. Briefly, the iron oxide nanoparticles coated either with gelatin (which provides hydroxyl and primary amine functional groups) or with dextran (which provides hydroxyl groups) were functionalized with activated double bonds by interacting the functional groups of the gelatin or dextran with excess divinyl sulfone (DVS) via the Michael addition reaction. The residual activated double bonds were then used for covalent binding of the different neurotrophic factors, again via the Michael addition reaction. The concentration of each neurotrophic factor conjugated to the nanoparticles was determined by using the appropriate ELISA Kit (PeproTech Asia, Israel, and Boster Immunoleader, USA).

### 2.3. Comparative Stability Studies of Free versus Conjugated Neurotrophic Factors

- *Stability in Cell Cultures.* Free or conjugated neurotrophic factors were added once to the medium of dissociated DRG cultures (final concentration 10 ng/mL, each factor). The cultures were placed in a 5% CO_2_ incubator at 37°C for 9 days. The culture medium was not changed during the entire period of the experiment, and aliquots from it were collected at different points in time after cultivation. The concentrations of the residual factors in the aliquots were measured using the appropriate ELISA Kit.
- *Stability at *37°C* in Medium.* Samples of culture media containing free or conjugated neurotrophic factors (final concentration 10 ng/mL, each factor) were kept at 37°C. Aliquots were collected at different time points, and the concentration of the residual free and conjugated neurotrophic factors in the samples was measured as described above.

### 2.4. Culture Medium

The culture medium was prepared from 90% Dulbecco's modified eagle medium-nutrient mixture F-12 (DMEM-F12), 10% heat-denatured fetal calf serum (FCS), 6 g/L D-glucose, 2 nM glutamine, 25 *μ*g/mL gentamycin, and 50 ng/mL IGF-I (all purchased from Biological Industries, Israel).

### 2.5. NVR-Gel for Neuronal Cultivation

NVR-Gel (NVR Labs proprietary [32]) is composed of two main components: high molecular hyaluronic acid (HA, 3 × 10^6^ Da) and laminin, both known to be inherent elements of the extracellular matrix. For cell cultivation, HA of 1% (BTG Polymers, Israel) was diluted with culture medium to the concentrations of 0.3–0.5%. Laminin (Sigma) was then mixed with the diluted HA to a final concentration of 10 ng/mL. Free or conjugated neurotrophic factors (*β*NGF, GDNF, and FGF-2) were finally added, separately or together (10 ng/mL of each factor), to complete the NVR-Gel composition.

### 2.6. Preparation of Organotypic DRG Cultures

All the experiments were carried out and authorized by the local ethics committee recognized by the Israeli authorities for animal experimentation. Stationary organotypic DRG cultures were prepared from rat fetuses (gestational day 15, Lewis inbred, Harlan, Israel). Immediately after dissection, the isolated ganglia were cut with a McIlwain tissue chopper into small slices (of 400 *μ*m thickness) which were then seeded in 12 well-culture plates (either directly on the plastic or on glass cover slips) containing 0.75 mL of NVR-Gel (one explant each well). Cultures received free or conjugated neurotrophic factors (10 ng/mL final concentration, each factor separately) once in the gel at seeding and subsequently with the nutrient medium at each consecutive feeding. Monitoring of the DRG growth pattern (neuronal sprouting, establishment of cultures, and myelin formation) was done by daily phase contrast microscopic observations from 24 hours after setting the cultures onward.

Experiments aimed at examining the effect of neurotrophic factors on early neuronal sprouting; cultures were performed with 12 repetitions (12 wells) for each neurotrophic factor. During the first week in culture, ganglia which exhibited nerve outgrowth were counted.

Experiments aimed at examining the effect of neurotrophic factors on early onset and progressionof myelin were performed in triplicate for each neurotrophic factor. Cultures with myelinated fibers, which in phase contrast microscopyappear confined by two dark lines along both sides of the fiber (due to the optical property of the myelin sheaths lipids), were recorded and photographed. The myelinated cultures were fixed for histological and immunocytochemical staining as well as for Transmission Electron Microscopy (TEM).

### 2.7. Immunofluorescence of Myelinated DRG Cultures

After removal of the culture medium, the DRG cultures were washed with phosphate buffered salt solution (PBS) and fixed in 4% paraformaldehyde for 15 min and then washed again with PBS. The fixed cells were permeabilized with 0.1% of Triton X-100 in PBS and then immunoblocked (to avoid nonspecific staining) with a 1% bovine serum albumin (BSA) in PBS for 1 h at room temperature. The specimens were then double incubated with mouse antimyelin basic protein antibodies (MBP, Covance, Nr. SMI 94 R, 1: 250) to visualize the myelin and rabbit antineurofilament antibodies (NF, Novus Biologicals, 1: 500) to visualize the neurite outgrowth. The primary antibodies were diluted in 0.1% BSA and 0.05% Tween-20 in PBS (diluents buffer) and incubated with the specimens overnight at 4°C. After rinsing with 0.05% Tween-20 in PBS (wash buffer), the DRG specimens were incubated for 1 h at room temperature with the appropriate secondary antibodies: Alexa-Fluor-488-conjugated donkey anti-mouse IgG or Alexa-Fluor-594-conjugated donkey anti-rabbit IgG (Jackson ImmunoResearch, USA, 1: 800 in a diluent buffer). Finally, the samples were rinsed again with wash buffer and mounted with mounting medium (Immco Diagnostics, USA). Myelinated cultures were alternatively stained with Luxol Fast Blue. All of the images were observed with an Olympus IX70 microscope.

### 2.8. Transmission Electron Microscopy (TEM) Analysis

Organotypic DRG cultures, detached from the glass or the plastic substrates, were fixed in 2.5% glutaraldehyde, washed in Sorensen phosphate buffer 0.1 M (pH 7.4) with 1.5% sucrose, and then fixed in 2% osmium tetroxide for 2 h. After dehydration in ethanol, samples were cleared in propylene oxide and embedded in Glauerts' embedding mixture of resins consisting of equal parts of Araldite M and Araldite Harter, HY 964 (Merck, Darmstadt, Germany), containing 0.5% of the plasticizer dibutyl phthalate and 1-2% of the accelerator 964, DY 064 (Merck, Darmstadt, Germany). Transverse cross sections of 70 nm were obtained using an Ultracut UCT ultramicrotome (Leica, Wetzlar, Germany). Sections were then stained with uranyl acetate and lead citrate and examined by a JEM-1010 transmission electron microscope (JEOL, Tokyo, Japan) equipped with a Mega-View-III digital camera and a Soft-Imaging-System (SIS, Münster, Germany) for the computerized acquisition of the images.

### 2.9. Statistical Analysis

Statistical analysis was performed by Student's* t*-test. The results are expressed as mean ± standard deviation (SD). *P* < 0.05 was accepted as indicating statistical significance.

## 3. Results 

### 3.1. Long-Term Stability of Free versus Conjugated Neurotrophic Factors

The stability of the free and conjugated neurotrophic factors (GDNF, *β*NGF, and FGF-2) against various enzymes and inhibitors, existing in serum or secreted from cells, was examined in tissue cultures and in medium alone at 37°C.

**Figure 1:**
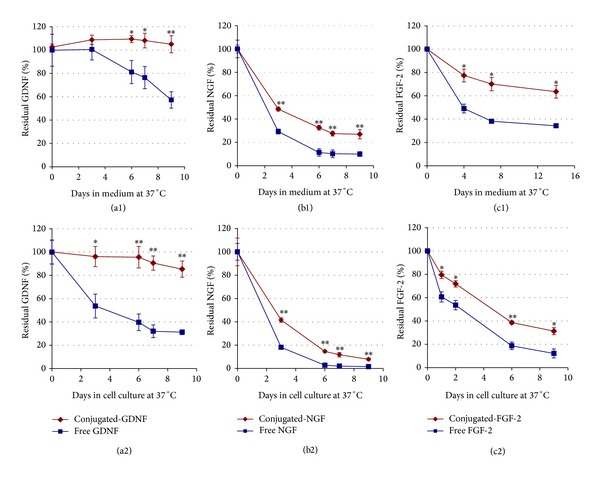
Stability of free versus conjugated neurotrophic factors at 37°C in the absence ((a1), (b1), and (c1)) and in the presence ((a2), (b2), and (c2)) of cells. In the upper row free or conjugated neurotrophic factors (GDNF, *β*NGF, and FGF-2) were added to culture medium, each type separately (10 ng/mL, final concentration), and placed at 37°C (in the absence of cells). Aliquots were collected after different time points, and the concentration of the residual factor in the samples was measured using an appropriate ELISA kit. In the lower row, the same concentrations of free or conjugated neurotrophic factors were added once to dissociate dorsal root ganglia (DRG) cell cultures at the beginning of the experiment. The culture medium was not changed during the experiment and aliquots from it were collected at different days after cultivation. The concentration of the residual factors in the aliquots was measured as described above. The data are presented as mean ± SD in triplicate (∗*P* < 0.01, ∗∗*P* < 0.001).

Results shown in Figure 1 demonstrate that the concentration of the neurotrophic factor decreased gradually over time; however, the conjugated neurotrophic factors were significantly more stable than the free factors in both medium alone ((a1)–(c1)) and tissue cultures ((a2)–(c2)). Among the conjugated factors, the most stable was the conjugated-GDNF ((a1) & (a2)), followed by the conjugated-FGF-2 with a moderate stability ((c1) & (c2)), and the least stable was the conjugated-*β*NGF ((b1) & (b2)). During the 9 days of the experiment, the concentration of the conjugated-GDNF remained almost stable at around 100%, while the concentration of free GDNF was gradually decreased to 57.3 ± 7.0% in medium and 31.2 ± 2.1% in culture. Regarding the stability of FGF-2, after about a week, the residual concentrations of the conjugated-FGF-2 in medium and in tissue culture were reduced to 70.1 ± 5.7% and 38.6 ± 1.4%, respectively. On the other hand, the residual concentrations of the free FGF-2 were significantly lower than that of the conjugated factor: 38.2 ± 1.6% and 18.7 ± 3.2%, in medium and in the tissue culture, respectively. As to the *β*NGF, after 6 days, the residual concentrations of the conjugated-*β*NGF were reduced to 32.6 ± 2.0% in medium and 14.6 ± 1.0% in culture. However, the free *β*NGF was decreased more significantly to 14.6 ± 1.0% in medium and 2.8 ± 1.5% in tissue culture.

### 3.2. Effects of Free versus Conjugated Neurotrophic Factors on DRG Organotypic Cultures

**Figure 2:**
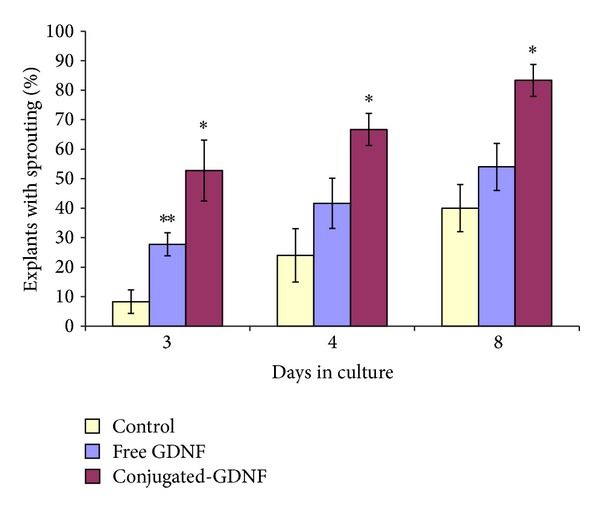
Enhancement of early nerve fibre outgrowth by conjugated-GDNF. The graph shows the percentage of explants (out of 12) which exhibited nerve fiber sprouting in the absence and the presence of free versus conjugated-GDNF as a function of time (days) in culture. The data are presented as mean ± SD from 3 repeated experiments. ∗ Significant difference (*P* < 0.05) compared to the corresponding free group. ∗∗ Significant difference (*P* < 0.05) compared to the corresponding control group.

**Figure 3:**
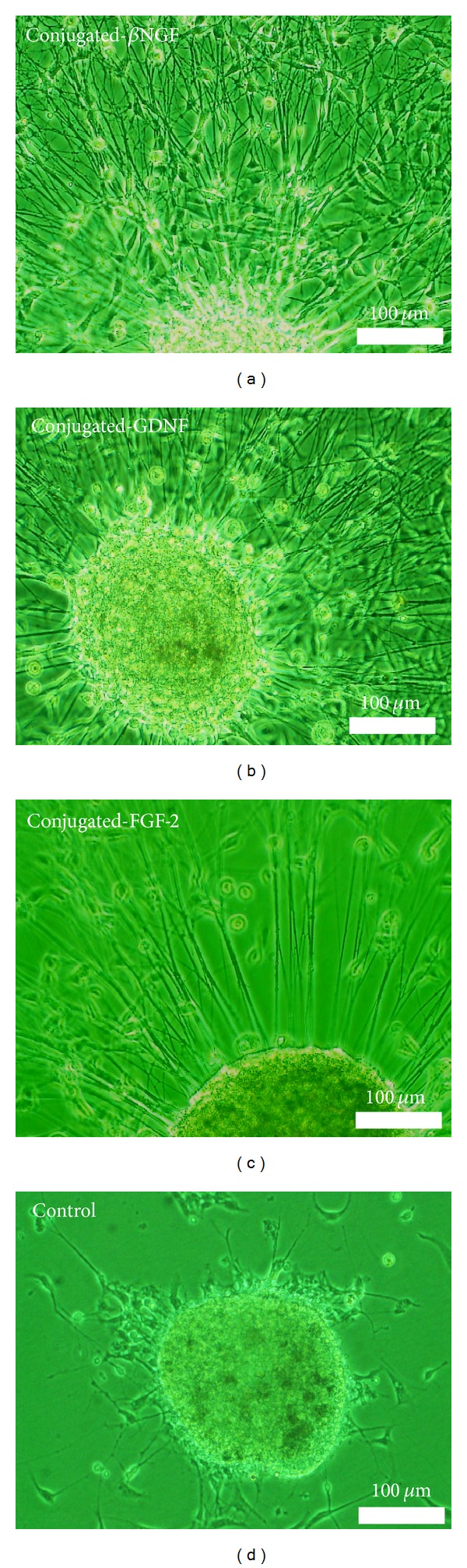
Early sprouting of thin and long nerve fibers from DRG explants 24 h after cultivation in NVR-Gel enriched with growth factors conjugated to iron oxide nanoparticles as follows: (a) Conjugated-*β*NGF, (b) Conjugated-GDNF, (c) Conjugated-FGF-2, (d) Control (without growth factors) (phase-contrast microscopy).

**Figure 4:**
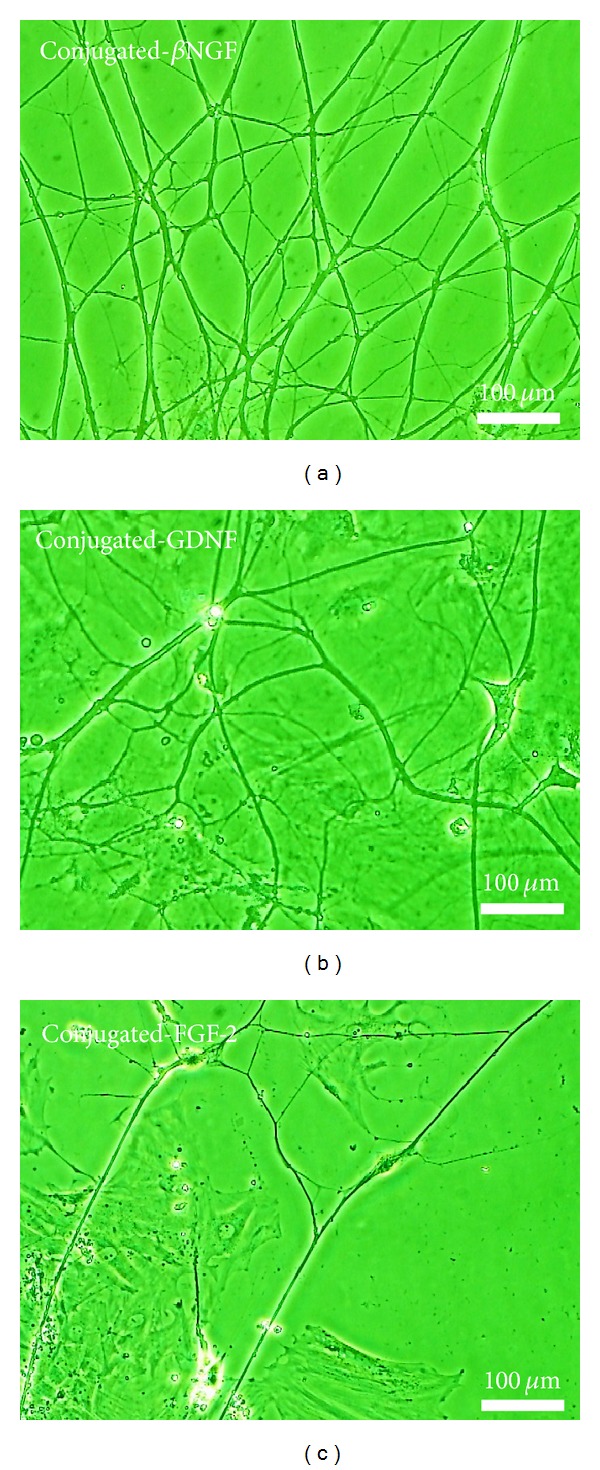
Establishment of cultures: neuronal networks composed of long, thick, and ramified neurites which are originating from DRG explants cultures, 10 days after set-up in NVR-Gel enriched with neurotrophic factors conjugated to iron oxide nanoparticles as follows: (a) Conjugated-*β*NGF, (b) Conjugated-GDNF, (c) Conjugated-FGF-2 (phase-contrast microscopy).

The overall effect of the exposure of DRG explants to each of the three conjugated neurotrophic factors is represented by the early phenomenon of sprouting compared to cultures that were exposed to free factors and control. During the first days of cultivation, the number of DRG explants that exhibited an early sprouting, following the exposure to conjugated-GDNF, was increased significantly by 30% compared to free GDNF and by 40% compared to controls (without neurotrophic factors) (Figure 2). The most intensive sprouting was observed in cultures which were exposed to *β*NGF and GDNF (Figure 3). During the establishment of the cultures (after the first week of cultivation), the regenerated nerve fibers became longer, thicker, and ramified, until the formation of neuronal networks, mainly in cultures exposed to conjugated-GDNF and *β*NGF (Figure 4). However, only conjugated-GDNF accelerated significantly the onset of myelin.

**Figure 5:**
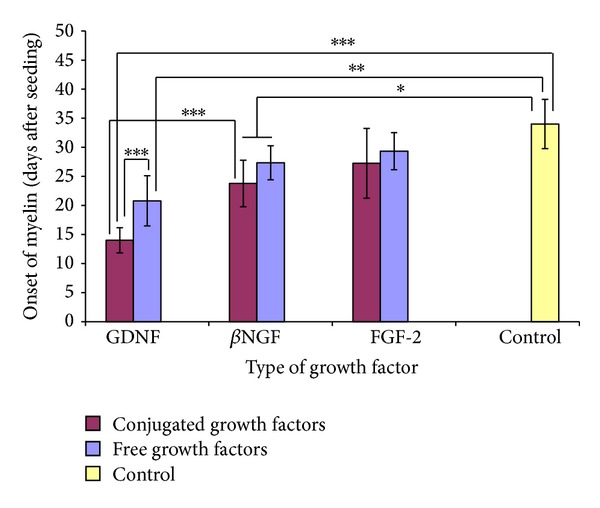
Effect of neurotrophic factors conjugated to iron oxide nanoparticles versus free factors on the onset of myelin in DRG organotypic cultures. The graph shows time elapsed (days) until the appearance of the first segments of myelinated axons. Control cultures have not been exposed to neurotrophic factors. The data represented shows mean ± SD from 15 repeated experiments. In each experiment cultures were performed in triplicate for each type of neurotrophic factor (∗*P* < 0.05, ∗∗*P* < 0.01, ∗∗∗*P* < 0.001).

**Figure 6:**
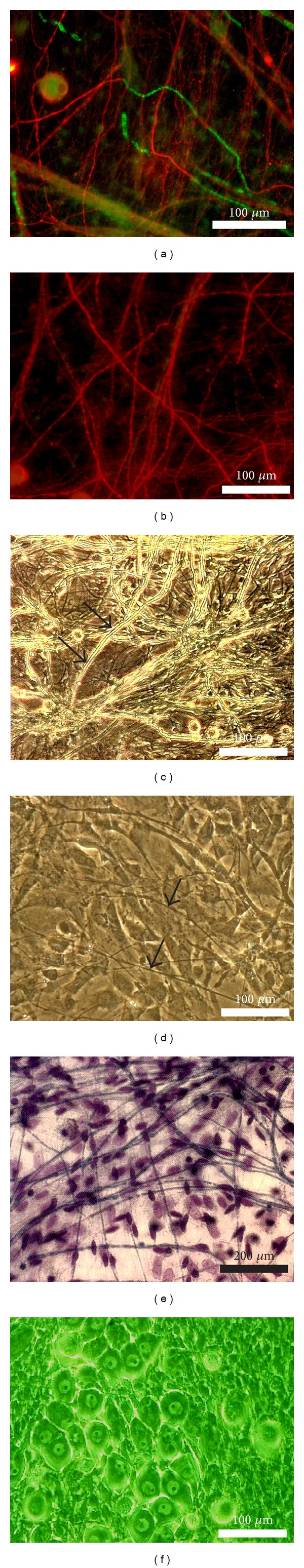
Myelinated organotypic DRG cultures exposed to conjugated-GDNF (left column) versus nonmyelinated cultures (right column). (a) and (b): Representative immunofluorescence images of a myelinated (day 12) versus nonmyelinated cultures, respectively. Neurites appear in red (antineurofilament staining) and myelin sheaths appear in green (antimyelin basic protein staining). (c) and (d): Phase-contrast microscope images of a heavily myelinated (day 16) versus nonmyelinated culture, respectively. Note the arrows in (c) which point to a single birefringent myelinated nerve fibre (appears as a bright fibre confined by two dark lines along both its sides due to the optical property of the myelin sheaths lipids). The arrows in (d) point to nonmyelinated fibres. (e): Staining of myelinated nerve fibres with Luxol Fast Blue. (f): Phase-contrast microscope image (through a green filter) of individual DRG cells in a nonmyelinated culture.

The effect of free versus conjugated neurotrophic factors on the onset of myelin in organotypic DRG cultures is shown in Figure 5. The results demonstrate that the onset of myelin (the appearance of initial segments of myelinated axons) was observed first in cultures exposed to conjugated-GDNF. In these cultures, onset of myelin was already observed after 14 ± 2.2 days of cultivation. Furthermore, during subsequent days, most of the fibers in cultures incubated with conjugated-GDNF became heavily myelinated (Figure 6). In contrast, the onset of myelin in cultures exposed to free GDNF was observed significantly later, that is, after 20.8 ± 4.3 days of cultivation (*P* = 0.0006, compared to conjugated-GDNF). The onset of myelin in cultures exposed to free and conjugated-*β*NGF was observed only after more than 3 weeks of cultivation (on days 27.3 ± 2.9 and 23.8 ± 4.0, resp.). Although in these cultures the onset of myelin was advanced (*P* < 0.05) in comparison to control cultures (without neurotrophic factors), yet it was significantly decelerated (*P* < 0.001) compared to cultures exposed to conjugated-GDNF. The onset of myelin in cultures exposed to free and conjugated-FGF-2 was observed around the fourth week in culture, on days 29.3 ± 3.2 and 27.3 ± 6.0, respectively, which demonstrates a nonsignificant (*P* > 0.05) acceleration compared to controls, in which the phenomenon occurred 34.0 ± 4.2 days after cultivation. Results similar to those obtained from control cultures were also obtained in cultures which were exposed to naked iron oxide nanoparticles.

### 3.3. TEM Analysis

**Figure 7:**
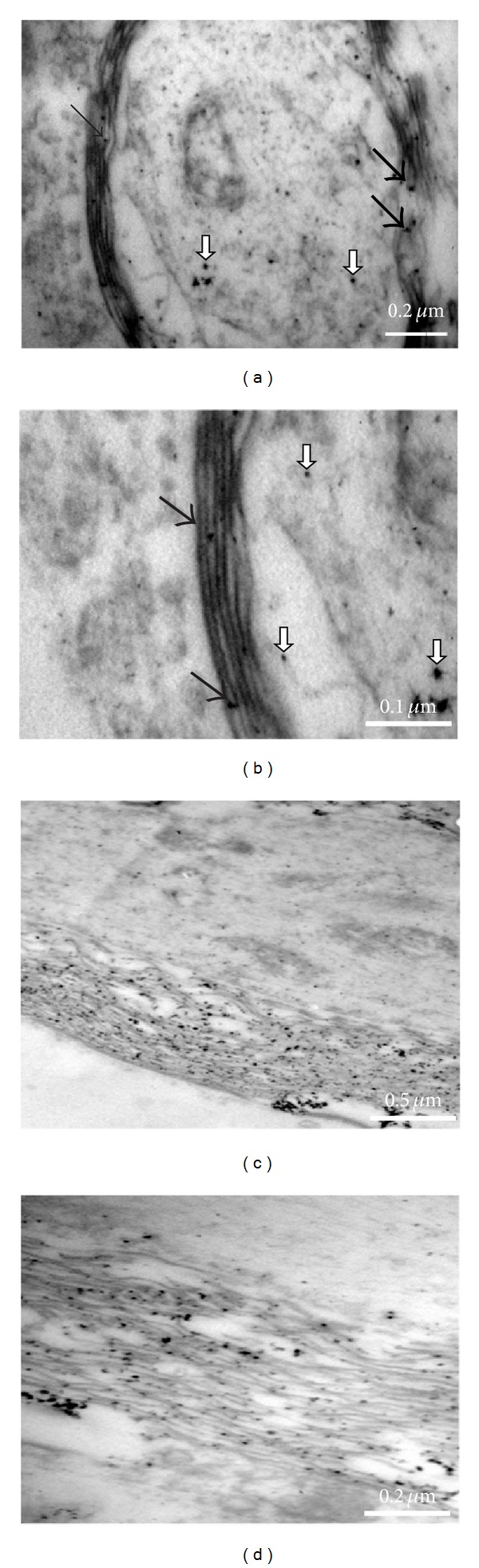
TEM analysis of the localisation of GDNF-conjugated iron oxide nanoparticles: Myelinated organotypic DRG cultures 14 days after seeding in NVR-Gel containing GDNF-conjugated nanoparticles were analysed. (a) and (b): Axon in transverse section. (c) and (d): Axon in longitudinal section. The black arrows point to iron oxide nanoparticles between myelin lamellae and the white arrows point to iron oxide nanoparticles in the axon.

TEM analysis was made on early myelinated DRG organotypic cultures (14 days after seeding) performed in NVR-Gel enriched with conjugated-GDNF. TEM observations (Figure 7) confirmed the light microscopic observations regarding the existence of myelin around the nerve fibers. However, TEM analysis allowed further observation of the very small single iron oxide nanoparticles and to determine their localization. In Figure 7, a transverse section ((a) and (b)) and a longitudinal section ((c) and (d)) of a nerve fiber with its myelin sheaths are shown. The ultrastructural analysis allowed us to observe single/distinct iron oxide nanoparticles (of average size of around 10–15 nm) localized between the ensheathing myelin lamellae (black arrows) and within the axon (white arrows). These TEM observations clearly demonstrate that the GDNF-conjugated iron oxide nanoparticles were internalized by neurons and by Schwann cells.

## 4. Discussion

The present study describes a novel strategy for DRG cultivation in NVR-Gel containing neurotrophic factors (*β*NGF, GDNF, and FGF-2) covalently conjugated to iron oxide nanoparticles. The neurotrophic factors were stabilized by the covalent conjugation to the iron oxide nanoparticles. This was demonstrated by comparative stability studies (Figure 1) in which the conjugated neurotrophic factors were significantly more stable than the free factors in cell cultures and medium at 37°C. The increased stability of the conjugated neurotrophic factors is probably due to the fact that the coupling of proteins to nanoparticles protects them from proteolytic enzymes and inhibitors existing in the serum or secreted by the cells and thus prolongs their half-life time and enhances their activity* in vitro* and* in vivo, *as described previously [21, 22, 33, 35–39].

The biological activity of the neurotrophic factors was examined in cultures prepared in NVR-Gel. The results showed that the gel adhered easily the DRG slices to the glass or plastic substrates and subsequently supported cell maturation and nerve fiber outgrowth in a three-dimensional pattern. Similar properties of the gel have been demonstrated previously, both in neuronal cell cultures [21, 40] and in* in vivo* models of peripheral nerve injuries and spinal cord injuries [41, 42].

Comparative studies on the biological effect of free versus conjugated-*β*NGF, -GDNF, and -FGF-2 on DRG cultures revealed that during the first days of cultivation the conjugated neurotrophic factors had more beneficial effects than the corresponding free neurotrophic factors on the enhancement of early nerve fiber regeneration (Figure 2). In the long-term cultures, the conjugated-GDNF had the most significant efficacy (Figure 5), which was expressed in acceleration of the myelin onset and subsequently the progression of myelination. This is probably due to covalent binding of the GDNF to the iron oxide nanoparticles, which increases the conjugated neurotrophic factor stability and prolongs its biological activity. As far as we know, there are no other publications who report that GDNF conjugated to nanoparticles, of any kind, enhances onset and progression of myelin. Free GDNF had a significantly reduced effect in comparison to the conjugated-GDNF.

The difference in the effect of conjugated-GDNF and conjugated-*β*NGF on the acceleration of myelin formation in DRG cultures is intriguing since both conjugated factors had a similar beneficial effect on early neurite outgrowth and formation of axonal networks. Since myelin of the peripheral nervous system is known to be formed only around axons that have a diameter of 2 *μ*m and more, we expected that myelin will equivalently form around the thick axons in cultures incubated with either of neurotrophic factors. However, the conjugated-GDNF accelerated the early onset and progression of myelin significantly over the conjugated-*β*NGF (*P* < 0.0001) (Figure 5). This strongly indicates that conjugated-GDNF, but not the *β*NGF, has an additional beneficial effect on Schwann cells, which results in the enhancement of the peripheral myelin formation.

Indeed, the TEM images (Figure 7) clearly demonstrate that a large number of iron oxide nanoparticles conjugated to GDNF were internalized by neurons as well as by Schwann cells. These results strongly support the hypothesis that GDNF activates both on DRG neurons and Schwann cells. This hypothesis is strengthened by Zhang et al. [11] who reported that free GDNF, beside its effect on enhancement of axonal regeneration* in vivo* and* in vitro, *significantly increases the number of myelin sheaths produced by Schwann cells. Similarly, Höke et al. [10] found that administration of free GDNF to adult rats alters axon-Schwann cell units and promotes myelination of unmyelinated nerve fibers.

## 5. Conclusion

These studies demonstrate that the covalent conjugation of neurotrophic factors to iron oxide nanoparticles increases their stability, preserves their activity, and even improves it. Comparative biological studies performed on DRG cultures in NVR-Gel revealed that the gel enriched with conjugated-*β*NGF, -GDNF, and -FGF-2 induced early nerve fiber regeneration significantly before the corresponding free neurotrophic factors. Among the three conjugated neurotrophic factors, the conjugated-GDNF had the most meaningful effect on the early onset and the progression of myelin in DRG cultures. The onset of myelin in cultures enriched with conjugated-GDNF was detected 7 days earlier than in cultures incubated with free GDNF and 21 days earlier than in control cultures.

Our culture strategy, which combines the employment of NVR-Gel and stabilized conjugated-GDNF, reveals that the biomaterials evaluated could become a scaffold material for a bioartificial nerve graft for long gap peripheral nerve reconstruction. Such an implant will most likely enhance regeneration and myelin formation of the damaged axons and thus has potential to shorten the recovery period. The organotypic DRG cultures in NVR-Gel enriched with conjugated factors can also serve as an* in vitro* pharmacological model for the research of demyelination and remyelination in autoimmune diseases and in bacterial and viral intoxications.
